# Binning Metagenomic Contigs Using Contig Embedding and Decomposed Tetranucleotide Frequency

**DOI:** 10.3390/biology13100755

**Published:** 2024-09-24

**Authors:** Long Fu, Jiabin Shi, Baohua Huang

**Affiliations:** 1 School of Computer and Electronic Information, Guangxi University, Nanning 530004, China; 2213393007@st.gxu.edu.cn (L.F.); 2213301041@st.gxu.edu.cn (J.S.); 2 Guangxi Key Laboratory of Digital Infrastructure, Guangxi Zhuang Autonomous Region Information Center, Nanning 530004, China

**Keywords:** metagenomics, binning, BERT, tetranucleotide, non-negative matrix factorization (NMF), Approximate Nearest Neighbors Oh Yeah (Annoy), DBSCAN algorithm

## Abstract

**Simple Summary:**

Metagenomic binning is a part of the metagenomic analysis process that can classify the genomes in complex microbial communities into possible source species. It is dedicated to restoring more complete genomes and is conducive to studying microbial community structure and function. Obtaining more effective feature representations of genome sequences to improve binning is still a major challenge. This study proposes a new metagenomic binning method called CedtBin, which uses an improved BERT model to obtain the embedded representation of contigs and splices it with the decomposed tetranucleotide frequencies into a new feature representation. Then, the Annoy-DBSCAN clustering algorithm is proposed, which can adaptively determine the parameters of the DBSCAN clustering algorithm for binning. The results show that CedtBin can achieve good binning effects on both simulated and real datasets, and can reconstruct more genomes.

**Abstract:**

Metagenomic binning is a crucial step in metagenomic research. It can aggregate the genome sequences belonging to the same microbial species into independent bins. Most existing methods ignore the semantic information of contigs and lack effective processing of tetranucleotide frequency, resulting in insufficient and complex feature information extracted for binning and poor binning results. To address the above problems, we propose CedtBin, a metagenomic binning method based on contig embedding and decomposed tetranucleotide frequency. First, the improved BERT model is used to learn the contigs to obtain their embedding representation. Secondly, the tetranucleotide frequencies are decomposed using a non-negative matrix factorization (NMF) algorithm. After that, the two features are spliced and input into the clustering algorithm for binning. Considering the sensitivity of the DBSCAN clustering algorithm to input parameters, in order to solve the drawbacks of manual parameter input, we also propose an Annoy-DBSCAN algorithm that can adaptively determine the parameters of the DBSCAN algorithm. This algorithm uses Approximate Nearest Neighbors Oh Yeah (Annoy) and combines it with a grid search strategy to find the optimal parameters of the DBSCAN algorithm. On simulated and real datasets, CedtBin achieves better binning results than mainstream methods and can reconstruct more genomes, indicating that the proposed method is effective.

## 1. Introduction

Metagenomics is an emerging research method that avoids the difficulties of traditional microbial culture techniques. It can sequence all the genetic information extracted from environmental samples, providing a new way to study microbial communities [1]. Microbial communities are inextricably linked to human diseases. When microbial communities are affected, it can lead to the occurrence of certain diseases such as asthma [2], allergies [3], autoimmune diseases [4], etc. The application of metagenomics to the microbiology field can help us better understand the relationship between microbial communities and human diseases and provide prevention or treatment tools to improve human health. Metagenomic binning is a critical step in metagenomics that allows genome sequences from the same microorganism to be placed into a bin, reconstructing a more complete genome and playing a pivotal role in analyzing the diversity and function of microbial communities.

Current metagenomic binning methods can be divided into three categories based on the characteristics of the information used for selection: nucleotide frequency-based, abundance-based, and nucleotide frequency- and abundance-based. Nucleotide frequency-based binning uses the similarity of nucleotide frequencies in genome sequences of the same species for binning. TETRA [5] calculates the tetranucleotide frequencies of the DNA sequences, then calculates their Pearson correlation coefficients, and uses this information for binning. CompostBin [6] uses a weighted PCA method for nucleotide frequency, which maps the data information into a low-dimensional space and reduces the dimensionality of the space. Differences in species abundance can lead to poor binning results for the two binning tools mentioned above. AbundanceBin [7] also uses nucleotide frequency but uses a separate Poisson distribution to model the reads obtained from sequencing, which can classify short sequences sampled from species with different abundances and achieve higher binning accuracy. MetaClusterTA [8], a tool for annotating metagenomic data, introduces binning technology and annotates the binning results based on the composition of tetranucleotides, resulting in more accurate and efficient annotations.

Abundance refers to the number of microbial species present in the environment. The abundance of each species in a metagenomic sample is different, and genome sequences from the same species have similar abundance characteristics. Genome sequences can be effectively classified based on this characteristic when the number of samples is large. Common binning methods based on abundance features include MetaGen [9] and Canopy [10]. MetaGen uses the relative abundance information of multiple samples to cluster contigs into different bins and relies on the Bayesian Information Criterion (BIC) to determine the number of genes in the sample. Canopy uses co-abundance information from multiple samples to reconstruct high-quality microbial genomes.

The binning method based on nucleotide frequency and abundance features combines the above two features, has a better binning effect, and is currently the most commonly used method in the field of metagenomic binning. Currently, such methods mainly include CONCOCT [11], MetaBAT2 [12], Maxbin2.0 [13], BMC3C [14], VAMB [15], CLMB [16], AVAMB [17], SemiBin [18], and GraphMB [19]. CONCOCT uses a Gaussian mixture model to cluster contigs based on nucleotide frequency and abundance features of multiple samples and applies an improved Bayesian model to automatically determine the number of clusters. MetaBAT2 uses empirical probability distances obtained from nucleotide frequency and abundance information and then uses graph clustering methods and label propagation algorithms for clustering. Maxbin2.0 also uses two features and the expectation maximization algorithm to estimate the probability that each sequence belongs to different bins. In addition to using nucleotide frequency and abundance information, BMC3C also added codon features for the first time, using an integrated method to optimize the binning results and dividing the different clustering results obtained multiple times into different subgraphs through a graph segmentation method, each of which represents a different bin. The VAMB method uses a variational autoencoder to encode nucleotide frequency and abundance features before clustering to obtain the potential representation of the DNA sequence and then uses an iterative medoid clustering algorithm to cluster it. CLMB uses deep contrastive learning to add simulated noise to the training data to improve the model’s feature learning ability for noisy data, achieving better robustness and stability. In contrast to VAMB, the AVAMB method uses an adversarial autoencoder to encode features and fuses VAMB. Although the computation time is longer, the number of nearly complete genomes reconstructed is greater. SemiBin uses a deep Siamese neural network to implement a semi-supervised method that uses the reference database and retains the ability to obtain high-quality binning without reference. GraphMB uses a graph neural network to integrate the assembly graph into the binning process. The combination of graph structure and sequence information improves the quality of metagenomic binning.

Although the use of nucleotide frequency and abundance information has apparent advantages, most methods do not pay attention to the complex semantic information, position information, and sequence length of the DNA sequence itself and lack the processing of nucleotide frequency. This will lead to limited and complex redundant feature information extracted for binning, making binning inefficient. Therefore, this study proposes CedtBin, a metagenomic binning method based on contig embedding and decomposed tetranucleotide frequency, which uses the mask training task of the BERT model to obtain the potential representation of contigs and splices it with the tetranucleotide frequency after non-negative matrix decomposition into a new feature for subsequent clustering tasks. The DBSCAN algorithm is improved, and the Annoy algorithm and grid search strategy are used to adaptively determine the two critical parameters Eps and MinPts. The experimental results show that CedtBin’s binning results on both simulated and real datasets, whether at the strain level, species level, or genus level, are significantly better than the current mainstream binning methods VAMB and MetaBAT2 and can reconstruct more genomes.

## 2. Materials and Methods

### 2.1. Datasets

In this study, the 2nd CAMI Toy Human Microbiome Project Dataset (CAMI2-HMP) [20] can be downloaded from the CAMI official website (https://data.cami-challenge.org, accessed on 18 July 2024). This simulated dataset includes five sub-datasets from five different parts of the human body: Airways (10 samples), Gastrointestinal (GI, 10 samples), Oral (10 samples), Skin (10 samples), and Urogenital (Urog, 9 samples). In addition, we also add a real dataset—MetaHIT (Metagenomics of the Human Intestinal Tract). MetaHIT is a vital human intestinal microbiome research project used to explore the composition and function of intestinal microorganisms and their relationship with human health and disease [21]. The MetaHIT dataset can be downloaded from https://portal.nersc.gov/dna/RD/Metagenome_RD/MetaBAT/Files (accessed on 18 July 2024). In contrast to the simulated dataset CAMI2-HMP, the real dataset MetaHIT contains various noises, increasing the data’s complexity and bringing challenges to the analysis. We discarded sequences with contig lengths of less than 2000 base pairs and only used the remaining contig sequences for the experiment. The details of the dataset are shown in Table 1.

### 2.2. Overview of CedtBin

**Figure 1:**
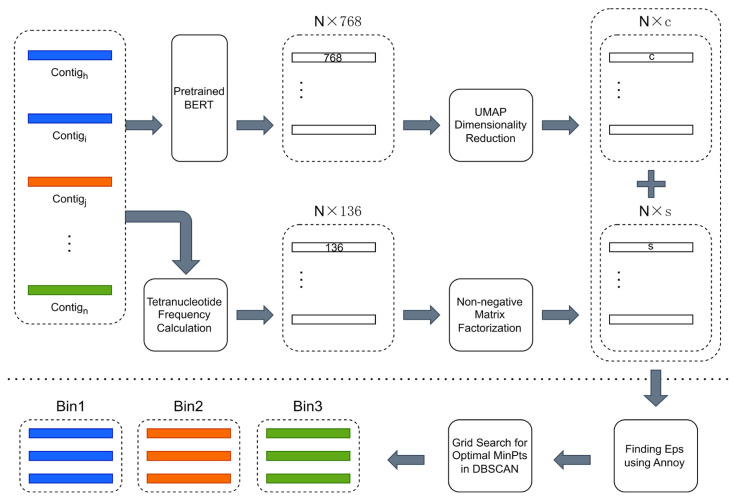
Overview of CedtBin workflow. The part above the long dashed line in the figure can be summarized as the feature acquisition stage, which involves two processes. First, the contigs are passed through the pre-trained BERT model and reduced in dimension to obtain an N × c contig embedding. Second, the contigs are directly calculated for tetranucleotide frequencies and decomposed using the NMF algorithm to obtain an N × s feature matrix. The part below the long dashed line is the clustering stage, where the two different features obtained in the previous stage are merged and fed into the clustering algorithm of this stage to obtain the final binning result.

The overview of CedtBin is shown in Figure 1. The N contig sequence data obtained by metagenomic sequencing are the input of CedtBin. Each contig will be processed twice to obtain two different feature matrices. The pre-trained BERT model encodes the contig into a 768-dimensional feature vector. After processing all contigs, a size N × 768 feature matrix can be obtained. Then, the matrix is reduced in dimension using the UMAP method to obtain an N × c feature matrix. After obtaining the embedded representation of all contigs in the metagenome, the tetranucleotide frequency of each contig is calculated to obtain an N × 136 feature matrix. Next, the NMF algorithm decomposes this matrix to obtain an N × s feature matrix. The two features are concatenated into a final feature matrix of size N × (s + c) and input into the Annoy algorithm to find the optimal parameter Eps. Then, the grid search strategy is combined to find another optimal parameter MinPts in the DBSCAN clustering algorithm and output the final binning result.

### 2.3. Contig Embedding

The BERT model uses the Transformer structure, which enables it to obtain contextual information of the sequence from left to right and from right to left, improving its ability to represent the semantics of the sentence [22]. BERT first performs unsupervised pre-training on a large-scale corpus, mainly including two tasks: masked language model and next sentence prediction. After pre-training, it can be fine-tuned with a small amount of labeled data on specific tasks to adapt it to various downstream NLP tasks. DNABERT [23] uses the BERT model to obtain a universal representation of DNA sequences for downstream tasks such as gene promoter prediction, splice site recognition, and transcription factor binding site identification. However, DNABERT is not used for metagenomic binning. We use the BERT model to learn contigs and obtain their universal representation for metagenomic binning tasks.

#### 2.3.1. Preprocessing

The contigs obtained from the metagenome are often of non-uniform length. Most contigs are between a few hundred base pairs and hundreds of thousands of base pairs in length, and the obtained k-mer is much larger than 512. Before pre-training, we need to pre-process the obtained data. We discard contigs with a base length of less than 2000 and use a sliding window method to k-merize the remaining contigs. In this study, the standard 4-mer of metagenome binning is used. 4-mer refers to a subsequence in a sequence with four bases. For example, a sequence ATTCGA has the following 4-mers: ATTC, TTCG, and TCGA.

There are 4^4^ = 256 different basic compositions for 4-mers, and these 256 different words are added to the dictionary. There are also five special characters in the dictionary. [CLS] is the first word in the sentence and can be used for subsequent classification tasks. [SEP] indicates the end of the sentence and is the last word in the sentence. [PAD] indicates a padding field. [MASK] indicates a masked word. [UNK] indicates an unknown word. At this point, we have created a dictionary with a total of 261 entries.

#### 2.3.2. Modified Mask Language Model

This study discards the next sentence prediction task and only uses the masked language model. Unlike human language text, which is composed of discrete words, k-mer can be inferred based on the information of the previous k-mer and the next k-mer. Therefore, the masking strategy needs to be modified to apply to contigs. Instead of masking only one k-mer in the original model, we mask a continuous piece of k-mer, which can better learn the latent semantic relationship of contigs.

For each k-mer in the input sequence, 15% of the k-mers are masked. If a k-mer is selected for masking, there are three processing methods:

- Eighty percent of the time, a continuous k-mer centered on the k-mer is replaced using [MASK];
- Ten percent of the time, a continuous k-mer centered on the k-mer is randomly replaced with another k-mer;
- Ten percent of the time, the k-mer remains unchanged.

In addition, we use a multi-scale masking strategy, in which k-mers of different scales (different masking lengths) are randomly selected for masking during each masking process. For example, some of the k-mers to be masked are shorter in length, while others are longer, so as to increase the model’s learning of different scales of context.

The masked training task uses cross-entropy loss as the loss function. During training, the parameters of the model are continuously adjusted according to the gradient of the loss so that it can understand and predict the masked k-mer information well according to the context. To improve the model’s learning ability, we use the ADAMW optimizer to adaptively adjust the learning rate during training.

For a contig, the contig is tokenized into 4-mers (a 4-mer is a token, corresponding to a unique integer ID in the dictionary) and divided into multiple different segments. A special token [CLS] is added in front of each segment, and a special token [SEP] is added at the end. The total length is 512, and the insufficiency is supplemented by special tokens [PAD]. Each segment is masked according to the above masking strategy and then passed through the embedding layer of the BERT model to obtain its word embedding representation and position embedding representation, and the sum of the two is used as the final embedding vector. In the mask training task, the embedding vector is input into the BERT model for training. The BERT model learns to predict these masked tokens based on the unmasked context tokens through a multi-layer Transformer network. The model calculates the loss by comparing the predicted tokens with the original tokens using the cross-entropy loss function. Then, backpropagation and parameter updates are performed based on this loss to optimize the model.

#### 2.3.3. Pooling

The contig is divided into several segments of 512 length. After the final representation of each segment passes through 12 Transformer layers, the [CLS] special character vector of the last hidden layer is used to represent the segment. Metagenome binning is for each contig, not for each segmented 512-length segment. To do this, it is necessary to perform an average pooling operation on these segments from the same contig to obtain the representation of the entire contig sequence.

### 2.4. Decomposed Tetranucleotide Frequency

#### 2.4.1. Tetranucleotide Frequency Calculation

Considering the reverse complementary characteristics of double-stranded genome sequences, there are only 136 different bases in the 256 base compositions obtained by tetranucleotide frequencies (i.e., 4-mers), effectively reducing the number of 4-mers. For example, ATGC and GCAT are reverse complementary and considered the same 4-mer. Therefore, a contig can be represented by a 136-dimensional feature vector:

(1) $$f=\left(f_{1},\;f_{2},\;\cdots ,\;f_{136}\right)$$

where $f_{j}$ is the cumulative number of occurrences of the *j*-th k-mer.

We then construct an N × 136-dimensional feature matrix T, where N is the number of contigs. To reduce the effect of the length of the contigs on the frequency of k-mers, we perform the following normalization operation:

(2) $$T_{i,j}=\frac{f_{i,j}}{\sum_{j=1}^{136}f_{i,j}}$$

where $f_{i,j}$ is the cumulative number of occurrences of the *j*-th k-mer in the *i*-th contig.

#### 2.4.2. Non-Negative Matrix Factorization

NMF is a matrix factorization technique that decomposes a non-negative matrix into the product of two or more non-negative matrices [24]. The tetranucleotide frequencies extracted in the previous section are the non-negative feature matrix T, and the transpose matrix is V = $T^{T}$. For a 136 × n non-negative matrix V, where n is the number of contigs, the NMF algorithm searches for k 136-dimensional basis vectors in this 136-dimensional real space, arranges them into a 136 × k basis matrix W, and projects the n 136-dimensional data onto the k 136-dimensional basis vectors to obtain a new set of n k-dimensional data, recorded as a k × n coefficient matrix H, such that the following formula holds:

(3) $$V_{136,n}=W_{136,k}\cdot H_{k,n}$$

The objective function of the NMF algorithm usually has Euclidean distance and Kullback–Leibler (KL) divergence. We use the Euclidean distance and the multiplication update rule to alternately update the base matrix W and the coefficient matrix H to gradually reduce the value of the objective function. The formula for the multiplication update rule is as follows:

(4) $$W_{136,k}\leftarrow W_{136,k}\frac{{\left(VH^{T}\right)}_{136,k}}{{\left(WHH^{T}\right)}_{136,k}}$$

(5) $$H_{k,n}\leftarrow H_{k,n}\frac{{\left(W^{T}V\right)}_{k,n}}{{\left(W^{T}WH\right)}_{k,n}}$$

After the update is completed, the coefficient matrix H replaces the original tetranucleotide frequency matrix V.

#### 2.4.3. Clustering

The DBSCAN algorithm [25] is a density-based clustering algorithm. Compared with other clustering algorithms, it can detect noise in sample data and find clusters of any shape and size in noisy sample data. It can achieve a better clustering effect in metagenomic binning. The principle of the DBSCAN algorithm is to use density connectivity to determine whether different data points belong to the same category. The DBSCAN algorithm does not require the size of the number of clusters to be specified in advance, but it has two key parameters that we need to set manually. One parameter is Eps, which represents the circular area centered on a given point; the other is MinPts, which means the minimum number of samples required within the circular area centered on a given point and having a radius of Eps. If the Eps value is set too large, it may cluster data from different categories together, resulting in a decrease in the number of bins. Conversely, if the Eps value is set too small, it may separate data that originally belonged to the same category into different clusters, leading to an increase in the number of bins. If the MinPts value is set too large, it will require more density to form clusters, resulting in a decrease in the number of bins. On the other hand, if the MinPts value is set too small, it may lead to incorrectly identifying low-density regions as clusters, producing a large number of small bins and resulting in an increase in the number of bins. Combining these two parameters will have a significant effect on the clustering results.

Annoy is a powerful and efficient algorithm for quickly finding approximate nearest neighbors in large datasets [26]. It is used in the music recommendations of the famous music application Spotify. The principle of Annoy is to use a random projection tree to build an index to quickly find the nearest point to the query point. Annoy sacrifices some accuracy to improve retrieval speed and can provide speedy query times on large datasets, much faster than brute force search.

We propose an Annoy-DBSCAN algorithm that can adaptively determine the parameters of the DBSCAN algorithm. This algorithm uses the Annoy algorithm and the grid search method to find the optimal parameters of the DBSCAN algorithm, which solves the drawbacks of manually entering parameters during the clustering process and can achieve better clustering results.

**Figure 2:**
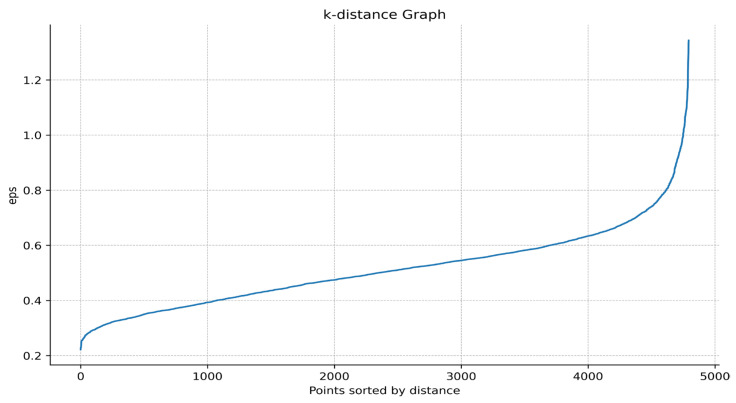
K-distance graph. The horizontal axis represents the data points sorted by distance, and the vertical axis represents the distance from the data point to the k-th nearest neighbor.

The implementation process of determining parameters using the Annoy-DBSCAN algorithm is described as follows:

i. Create an Annoy index. Add all data points to the index and then build a random projection tree.
ii. Calculate k-distance. For each data point, use the Annoy algorithm to find its k nearest neighbors and record the distance of the kth nearest neighbor. Store the k-distance values of all points in a list and sort them.
iii. Draw a k-distance graph. Use Matplotlib to draw a k-distance graph and select the inflection point from the graph as the Eps of the DBSCAN algorithm. For example, in Figure 2, it can be found that there is a sharp increase between the distance 0.6 and 0.9, so Eps can take the inflection point position here as 0.7.
iv. Grid search. After determining Eps, use the grid search method to find it in the preset MinPts value and select the optimal MinPts according to the clustering results.

## 3. Results

### 3.1. Experimental Instructions

The experiment uses the NC A100 v4 series virtual machine provided by the Microsoft platform, which is equipped with 1 NVIDIA A100 PCIe GPU, 80 GB GPU memory, and 24 non-multithreaded AMD EPYC Milan processor cores. The BERT-base model (total parameter size is 110 M) is used to pre-train the model using the Airways, Gi, Skin, and Urog datasets as training sets and the Oral dataset as the test set. During training, the batch size is set to 64, the initial learning rate is 2 × 10^−5^, and the multi-scale masking length is randomly selected from {1, 2, 4}, and other parameters are default. The ADAMW optimizer is used to optimize the training process, and a total of 10 epochs are trained.

**Figure 3:**
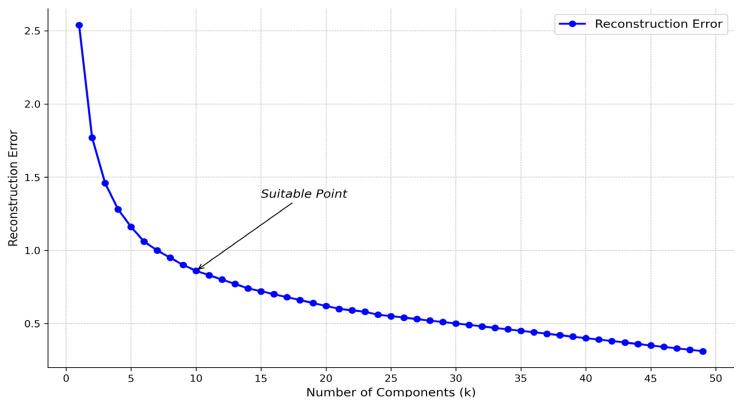
Relationship between k-value and reconstruction error.

When the NMF algorithm decomposes the original matrix V, the choice of k value is also critical. We construct the reconstruction error under different k values. Figure 3 shows that the reconstruction error decreases significantly slower when k is between about 6 and 15. In this appropriate range, k = 10 can be selected. In the following experiments, the k value is defaulted to 10. In addition, the NMF algorithm uses the initialization parameter init = nndsvd to accelerate the algorithm’s convergence and improve the results’ stability and quality.

Prior to feature concatenation, the dimension of the contig embedding features is reduced to 24 dimensions using the UMAP algorithm (https://umap-learn.readthedocs.io, accessed on 26 July 2024) to simplify the computation and improve efficiency. After concatenation, an N × 34 dimensional feature matrix is obtained for the clustering algorithm. In determining the parameters of the DBSCAN algorithm, the Annoy algorithm is used to perform an approximate nearest neighbor search. In order to balance query accuracy and efficiency, the number of trees to be built is set to n_trees = 15, and the distance between data is calculated using the Euclidean distance. The MinPts values for the grid search are {5, 10, 15, 20, 25}. Notably, unless otherwise specified, all experiments in this study use the Annoy-DBSCAN algorithm for the clustering process.

### 3.2. Results and Analysis of Different Features

#### 3.2.1. Evaluation Metrics

This study uses three metrics to measure the effectiveness of metagenomic binning: accuracy, recall, and F1 score. These metrics are commonly used in the field of metagenomic binning. Accuracy measures the ability of the binning method to correctly assign contigs to specific genomic bins. Specifically, it indicates the proportion of contigs that actually belong to a bin among all contigs predicted to be bins. A high accuracy means that most predictions are correct among the contigs predicted to be bins. Recall measures the ability of the binning method to identify all contigs that actually belong to a bin. Specifically, it indicates the proportion of all contigs that actually belong to a bin that are correctly assigned to that bin. A high recall means that the binning method is able to identify most of the contigs that actually belong to a bin. The F1 score is the harmonic mean of accuracy and recall, which takes into account the precision and recall of the classifier. It strikes a balance between accuracy and recall and is particularly suitable for situations where data distribution in metagenomics is uneven.

This study constructs a 2 × 2 confusion matrix to help calculate these indicators and classifies the binning results of contigs into four situations: TP, FP, TN, and FN. TP refers to the number of contigs that actually belong to a particular bin and are correctly assigned to that bin. FP refers to the number of contigs that do not actually belong to a specific bin but are incorrectly assigned to that bin. TN refers to the number of sequences that do not actually belong to a specific bin and are correctly identified as not belonging to that bin. FN refers to the number of contigs that actually belong to a bin but are incorrectly assigned to other bins. The calculation formulas for the three indicators are as follows:

(6) $$\mathrm{Precision}\;=\frac{\mathrm{TP}}{\mathrm{TP}+\mathrm{FP}}$$

(7) $$\mathrm{Recall}\;=\frac{\mathrm{TP}}{\mathrm{TP}+\mathrm{FN}}$$

(8) $$F1\;=\frac{2\times \mathrm{TP}}{2\times \mathrm{TP}+\mathrm{FP}+\mathrm{FN}}$$

#### 3.2.2. Binning Performance of Different Features

We perform the following ablation experiments to compare the performance of different features. Four features are compared: original tetranucleotide frequency (TNF), decomposed tetranucleotide frequency (Dec_TNF), contig embedding, and contig embedding + Dec_TNF. Their accuracy, recall, and F1 score are calculated at the species level on the five subsets of the CAMI2-HMP simulated dataset.

**Figure 4:**
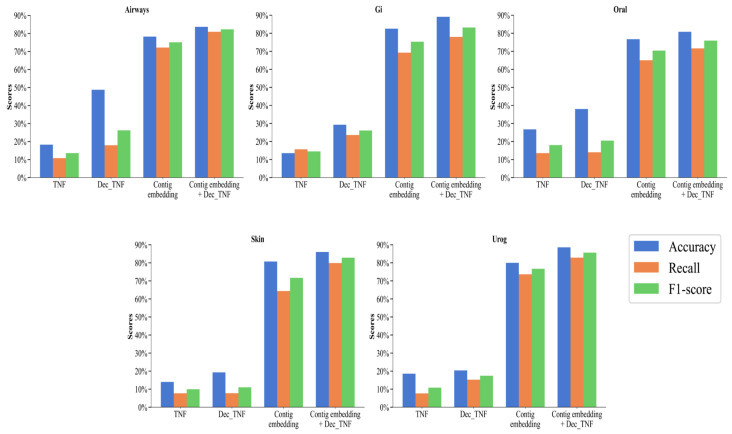
Binning performance of four features on simulated datasets at the species level.

As can be seen in Figure 4, on the five simulated datasets, the performance of the method using only TNF as a feature is the worst, with an accuracy of between 13.55% and 26.72%, a recall of between 7.65% and 15.68%, and an F1 score of between 9.95% and 18.02%. This is because unprocessed tetranucleotide frequencies can provide some local features of the sequence, but this type of information is relatively small and may not be sufficient to distinguish complex or similar genomes. Dec_TNF has a more significant improvement over TNF, with an accuracy rate from 19.28% to 48.74%, a recall rate from 7.74% to 23.59%, and an F1 score from 11.04% to 26.23%. NMF can extract the main components of the tetranucleotide frequencies, thereby capturing the main patterns in the sequence and making the features more compact and representative. In addition, NMF can reduce the noise and redundant information in the data, thereby improving the discriminability of the features.

The most significant is contig embedding with an accuracy between 76.70% and 82.52%, a recall between 64.39% and 73.65%, and an F1 score between 70.38% and 76.68%. All three indicators are far superior to TNF and Dec_TNF, with an increase in the F1 score of 60.43% and 53.54%, respectively. This fully proves that by using the BERT model to learn contigs and generate embedding vectors, these vectors can capture the context and semantic information of contigs, making the features more comprehensive, effective, and more accessible to distinguish between different genomes and improving the binning performance.

Contig embedding provides global context information of contigs, while Dec_TNF provides the main components of local patterns. The concatenation of contig embedding and Dec_TNF as features used by the CedtBin method can make it perform better on different types of contigs, thereby improving the overall performance. The accuracy of the concatenated features is between 80.79% and 89.12%, the recall rate is between 71.57% and 82.87%, and the F1 score is between 75.90% and 85.62%, which is improved in all indicators compared to the single use of contig embedding.

### 3.3. Results and Analysis of Different Binning Methods

#### 3.3.1. Simulated Dataset CAMI2-HMP Binning Results

To demonstrate the effectiveness of CedtBin in binning, the binning methods CedtBin, VAMB, and MetaBAT2 are compared on the simulated dataset CAMI2-HMP. The binning performance is measured by calculating the number of reconstructed near-complete (NC, recall > 90% and precision > 95%) genomes. Unlike the previous definitions, recall and precision are calculated in a way that takes into account the base pair coverage between the genome and the bins. Recall is the proportion of base pairs in a genome that are correctly assigned to a bin, and precision is the proportion of base pairs in a bin that are correctly assigned to a genome. Use the benchmark.py script in VAMB to obtain the number of reconstructed genomes.

**Figure 5:**
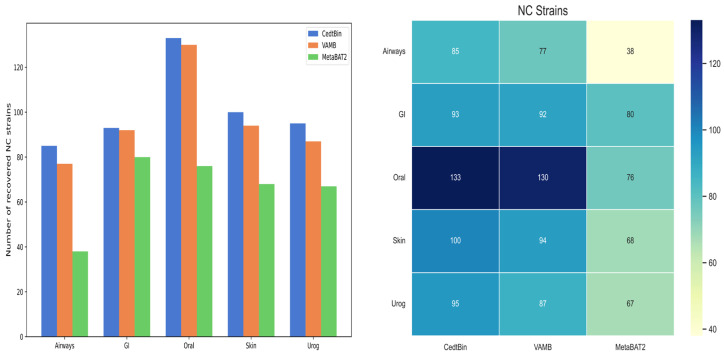
Number of reconstructed NC strains by three binners on simulated datasets. The left side of the figure is described as a bar chart, and the right side is described as a heatmap.

Binning methods VAMB and MetaBAT2 both use default parameters for metagenome binning. The number of NC genomes reconstructed from the strain level on the five datasets of Airways, GI, Oral, Skin, and Urog is shown in Figure 5, and the specific values are given in Table 2. Compared to VAMB and MetaBAT2, the CedtBin method performs best on all datasets, with the largest number of reconstructed NC genomes. On the Airways dataset, CedtBin reconstructs 85 NC genomes, significantly higher than VAMB’s 77 and MetaBAT2′s 38, an increase of approximately 10.39% over VAMB and 123.68% over MetaBAT2. On the GI dataset, CedtBin and VAMB perform similarly with 93 and 92, respectively, both better than MetaBAT2′s 80. On the Oral dataset, CedtBin reconstructs 133 NC genomes, slightly better than VAMB’s 130, but far better than MetaBAT2′s 76, an increase of 75%. On the Skin dataset, CedtBin reconstructs 100 NC genomes, which exceeds the 94 reconstructed by VAMB and the 68 reconstructed by MetaBAT2, with an improvement of 6.38% and 47.06%, respectively. On the Urog dataset, CedtBin performs best, reconstructing 95 NC genomes, which exceeds the 87 reconstructed by VAMB and the 67 reconstructed by MetaBAT2, with an improvement of 9.20% and 41.79%, respectively. In terms of the total number of NC genomes, CedtBin reconstructs 506 genomes, VAMB reconstructs 480, and MetaBAT2 reconstructs 329. Compared with VAMB, CedtBin’s improvement percentage is about 5.42%. Compared with MetaBAT2, CedtBin’s improvement percentage is about 53.80%.

In addition, CedtBin outperforms MetaBAT2 and VAMB at the species and genus level in most cases, also indicating that our method is more suitable for binning on these complex datasets. As shown in Table 3, CedtBin is able to reconstruct a greater number of genomes when reconstructing low-quality genomes with a reconstruction accuracy greater than 95% and a recall rate greater than 50% at the species level. Compared to MetaBAT2, CedtBin reconstructs 122 more genomes, an increase of 28.97%. Compared to VAMB, CedtBin reconstructed 51 more genomes, an increase of 10.37%. When the reconstruction accuracy is greater than 95% and the recall rate is 99%, CedtBin reconstructs more genomes than MetaBAT2 and VAMB. CedtBin reconstructs 67 and 36 more genomes than Maxbin2 and VAMB, respectively, an increase of 33.50% and 15.58%. Compared to the species level, the number of reconstructed genomes at the genus level is generally reduced, which is expected because the genus is a higher taxonomic level. As shown in Table 4, the CedtBin method reconstructs one to two fewer genomes than the VAMB method on the Oral dataset. In addition, in most cases, the CedtBin method still shows excellent performance at the genus level, reconstructing more genomes than the other two methods.

#### 3.3.2. Real Dataset MetaHIT Binning Results

**Figure 6:**
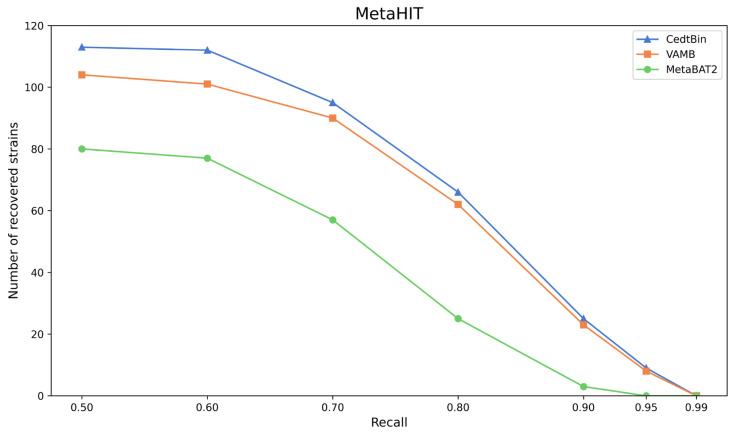
Number of reconstructed strains by three binners on the MetaHIT dataset.

The three methods CedtBin, MetaBAT2, and VAMB are run on the real dataset MetaHIT to obtain the number of genomes reconstructed with an accuracy greater than 95% at the strain level. The results are shown in Figure 6 and Table 5.

After analysis, we find that CedtBin performs better than VAMB and MetaBAT2 at all recall rates. In the range of 0.50 to 0.95 recall rates, CedtBin is able to reconstruct more genomes and shows more robust performance. At a recall rate of 0.5, CedtBin reconstructs 9 more genomes than VAMB and 33 more than MetaBAT2. At a recall rate of 0.9, CedtBin reconstructs 2 NC genomes more than VAMB and 22 NC genomes more than MetaBAT2. At a recall rate of 0.99, none of the methods reconstructs a genome, indicating that the task is challenging at this recall rate on real datasets.

### 3.4. Memory Usage and Runtime

To investigate how much memory and time the NMF and Annoy algorithms consume, we run experiments on the simulated dataset CAMI2-HMP and the real dataset MetaHIT (see Table 1 for dataset details) and record the running time and maximum memory consumption, as shown in Table 6. The column labeled “TNF” records the memory and time used for the entire binning process using only raw tetranucleotide frequency as the feature. The column “Dec_TNF” records the memory and time used for binning after first applying non-negative matrix factorization (NMF) to the tetranucleotide frequency (TNF) feature. “CedtBin” uses contig embedding and concatenated features from Dec_TNF, obtaining cluster representation on a pre-trained BERT model. It records the complete time and memory usage from obtaining encoded representations of the input sequences to clustering. The last two columns in the table represent the time and memory usage for the standalone clustering process using the DBSCAN algorithm and the Annoy-DBSCAN algorithm within CedtBin.

Using TNF to bin, the dataset dimension is N × 136, and it takes very little time, from 42.91 s to 97.96 s, and the maximum memory usage is the largest, from 9959.82 MiB to 55,506.55 MiB. This is because the features are not processed, resulting in high memory usage during clustering. Using Dec_TNF to bin, the dimension of the dataset is N × 10. Although it takes about 38.93 s more time on average than TNF (it takes some time to decompose the original feature matrix), it can be seen that the maximum memory usage is reduced from 3002.84 MiB to 5246.63 MiB, and the maximum memory usage is reduced by about 83.34% compared to TNF. This fully proves that the tetranucleotide frequency features processed by the NMF algorithm can effectively improve the binning effect and significantly reduce the memory usage. The dataset dimension of CedtBin is N × 34. Due to the addition of contig embedding, the time and memory requirements increase. The whole binning process takes between 28 min 38 s and 55 min 55 s. The maximum memory usage is on average about 2474.05 MiB more than Dec_TNF.

In CedtBin, we use DBSCAN and Annoy-DBSCAN for comparison. When using DBSCAN, we empirically fill in the two parameters Eps and MinPts and then run it. The average time used for this part of DBSCAN is 4.09 s, and the average maximum memory usage is 396.55 MiB. It can be seen that clustering using the DBSCAN algorithm is very fast when the feature dimension is small. Annoy-DBSCAN combines the Annoy algorithm with a grid search strategy to obtain these two parameters. The average time for the whole process is 28.44 s, and the average maximum memory usage is 488.49 MiB. Although the Annoy-DBSCAN method increases the average usage time by about 24.35 s and the average maximum memory usage by about 91.94 MiB compared to the DBSCAN method, 24.35 s is relatively insignificant in the entire CedtBin binning process. If manual parameter input can be avoided, this time sacrifice is worthwhile. In addition, we can also balance the search time and search accuracy by adjusting the parameters of the Annoy algorithm.

## 4. Discussion

This study proposes a new metagenomic binning method, CedtBin, which uses contig embedding and decomposed tetranucleotide frequency for binning. Metagenomic binning methods have been studied for many years, but little attention has been paid to the semantic information of contigs. The experimental results show that contig embedding is a very effective feature for metagenomic binning. Tetranucleotide frequency is the most commonly used feature, but the lack of processing of the original features leads to limited information and poor binning effect. It is optimized by the NMF algorithm. Although the improvement effect is limited, the processed features are more discriminative and significantly reduce time and memory. We chose the DBSCAN algorithm for clustering because it can cluster according to the density of the data and detect the noise in the data. It is suitable for metagenomic binning tasks and has been used in many studies. However, the DBSCAN algorithm requires manual input of the Eps and MinPts parameters and is very parameter sensitive. For this reason, we use the Annoy algorithm combined with a grid search strategy to adaptively determine these two parameters.

The CAMI2-HMP simulation dataset was chosen due to its role in providing a standardized benchmark for evaluating metagenomic binning methods [20], as CAMI was designed to offer a unified evaluation standard for binning performance and is widely used in the field. Simulation datasets generally have high-quality contigs and often result in good binning performance. However, real environments are more complex and variable, with poorer sequence quality, which can lead to differences in binning performance compared to simulation datasets. Although CedtBin performs better on the real MetaHIT dataset compared to other methods, it may face challenges in more complex environments. Therefore, it is necessary to train deep learning models on a broader range of datasets to enhance their applicability to real metagenomic data.

CedtBin has achieved good binning results in metagenome binning, but some work needs to be done. First, research on the BERT model is limited due to limited resources and performance constraints. In terms of masking strategies, in the future, the number of masked k-mers can be changed from 15% to 40%, and the effect of different masking lengths can be explored. Secondly, in terms of feature selection, adding features such as codons, GC content, and abundance can be considered. It is believed that the addition of these features can further improve the effect of binning.

## 5. Conclusions

This study proposes a new metagenomic binning method based on contig embedding and decomposed tetranucleotide frequency—CedtBin. The improved BERT model is used to obtain the embedding representation of the contigs and splices together with the tetranucleotide frequencies after non-negative matrix decomposition and then fed into the Annoy-DBSCAN clustering algorithm to obtain the clustering results. Annoy-DBSCAN is a clustering algorithm proposed in this study that can adaptively determine the parameters of the DBSCAN algorithm. The algorithm uses the Annoy algorithm and grid search method to find the optimal parameters of the DBSCAN algorithm, which solves the drawback of manually entering parameters during the clustering process and can achieve better clustering results. Whether in simulated datasets or real datasets, the performance of the CedtBin method is better than the mainstream binning methods VAMB and MetaBAT2, and it can reconstruct more genomes.

## Figures and Tables

**Table 1:** Detailed information of the dataset.

Datasets	Samples	Genus	Species	Strains	Contigs	N50
Airways	10	139	311	639	187,685	28,290
Gi	10	63	141	250	81,602	701,026
Oral	10	107	249	673	201,563	25,427
Skin	10	106	247	531	173,927	66,557
Urog	9	47	118	234	57,762	877,598
MetaHIT	264	70	219	291	179,534	8461

**Table 2:** Number of genomes at the strain level reconstructed with a precision of at least 95%.

		RECALL						
Dataset	Binner	0.50	0.60	0.70	0.8	0.9	0.95	0.99
Airways	MetaBAT2	82	73	68	57	38	31	21
	VAMB	126	123	118	110	77	58	42
	CedtBin	**142**	**135**	**131**	**119**	**85**	**66**	**48**
Gi	MetaBAT2	99	96	94	89	80	68	59
	VAMB	114	112	110	108	92	**86**	70
	CedtBin	**133**	**130**	**128**	**115**	**93**	**86**	**71**
Oral	MetaBAT2	86	84	81	79	76	61	45
	VAMB	180	**170**	**164**	**156**	130	110	82
	CedtBin	**183**	168	163	**156**	**133**	**113**	**84**
Skin	MetaBAT2	105	98	93	77	68	57	48
	VAMB	140	139	136	119	94	79	62
	CedtBin	**154**	**150**	**143**	**125**	**100**	**85**	**73**
Urog	MetaBAT2	75	73	71	69	67	63	45
	VAMB	118	113	106	99	87	74	51
	CedtBin	**132**	**122**	**115**	**105**	**95**	**83**	**58**

**Table 3:** Number of genomes at the species level reconstructed with a precision of at least 95%.

		RECALL						
Dataset	Binner	0.50	0.60	0.70	0.8	0.9	0.95	0.99
Airways	MetaBAT2	78	71	64	55	39	30	19
	VAMB	103	100	96	90	61	43	28
	CedtBin	**105**	**104**	**99**	**92**	**65**	**47**	**32**
Gi	MetaBAT2	92	88	87	83	76	67	57
	VAMB	81	80	80	80	71	68	57
	CedtBin	**111**	**105**	**104**	**100**	**85**	**81**	**68**
Oral	MetaBAT2	85	84	79	76	68	57	40
	VAMB	129	**124**	**121**	**117**	**98**	81	59
	CedtBin	**130**	**124**	119	116	97	**82**	**60**
Skin	MetaBAT2	101	95	91	75	65	53	44
	VAMB	108	107	104	90	66	58	47
	CedtBin	**115**	**111**	**109**	**101**	**79**	**63**	**55**
Urog	MetaBAT2	65	62	61	61	58	55	40
	VAMB	71	71	68	66	59	55	40
	CedtBin	**82**	**79**	**73**	**69**	**65**	**60**	**52**

**Table 4:** Number of genomes at the genus level reconstructed with a precision of at least 95%.

		RECALL						
Dataset	Binner	0.50	0.60	0.70	0.8	0.9	0.95	0.99
Airways	MetaBAT2	49	44	43	35	24	17	9
	VAMB	55	53	51	**49**	30	17	10
	CedtBin	**57**	**56**	**53**	**49**	**34**	**23**	**13**
Gi	MetaBAT2	**56**	54	**52**	45	39	32	28
	VAMB	45	44	44	44	37	35	32
	CedtBin	55	**55**	**52**	**48**	**43**	**40**	**35**
Oral	MetaBAT2	57	54	53	48	44	38	27
	VAMB	**65**	**63**	**62**	**61**	53	46	**36**
	CedtBin	63	62	60	60	**55**	**47**	35
Skin	MetaBAT2	65	63	62	53	48	39	34
	VAMB	55	55	54	51	39	34	28
	CedtBin	**68**	**65**	**64**	**62**	**53**	**45**	**39**
Urog	MetaBAT2	33	31	31	30	27	25	21
	VAMB	31	31	31	29	25	24	19
	CedtBin	**35**	**33**	**32**	**30**	**29**	**29**	**23**

**Table 5:** Number of genomes at the strain level reconstructed with a precision of at least 95% on the MetaHIT dataset.

		RECALL						
Dataset	Binner	0.50	0.60	0.70	0.8	0.9	0.95	0.99
MetaHIT	MetaBAT2	80	77	57	25	3	0	0
	VAMB	104	101	90	62	23	8	0
	CedtBin	**113**	**112**	**95**	**66**	**25**	**9**	0

**Table 6:** Runtime and peak memory usage on datasets.

Dataset		TNF	Dec_TNF	CedtBin	DBSCAN of CedtBin	Annoy-DBSCAN of CedtBin
Airways	Time	1 min 31 s	2 min 18 s	55 min 55 s	5.00 s	38.73 s
	Memory	55,506.55 MiB	5246.63 MiB	8680.34 MiB	628.23 MiB	725.45 MiB
Gi	Time	1 min 4 s	1 min 12 s	45 min 54 s	3.36 s	22.14 s
	Memory	9959.82 MiB	3167.38 MiB	5813.71 MiB	206.89 MiB	316.33 MiB
Oral	Time	1 min 33 s	3 min 2 s	51 min 38 s	5.17 s	35.56 s
	Memory	32,263.70 MiB	4292.60 MiB	6917.91 MiB	516.80 MiB	617.94 MiB
Skin	Time	1 min 38 s	2 min 15 s	51 min 38 s	5.49 s	33.35 s
	Memory	41,357.69 MiB	5744.48 MiB	8236.53 MiB	556.55 MiB	671.12 MiB
Urog	Time	42.91 s	45.81 s	28 min 38 s	0.80 s	8.13 s
	Memory	13,101.41 MiB	3002.84 MiB	4082.00 MiB	80.91 MiB	109.49 MiB
MetaHIT	Time	50.54 s	1 min 41 s	46 min 37 s	4.75 s	32.72 s
	Memory	49,518.02 MiB	4245.40 MiB	6813.13 MiB	389.90 MiB	490.63 MiB

## Data Availability

The CAMI2-HMP datasets can be downloaded from the CAMI official website (https://data.cami-challenge.org, accessed on 18 July 2024). The MetaHIT dataset can be downloaded from https://portal.nersc.gov/dna/RD/Metagenome_RD/MetaBAT/Files (accessed on 18 July 2024). CedtBin is open-source and available at https://github.com/gxulf/CedtBin (accessed on 26 July 2024).
